# *Helicobacter pylori* GmhB enzyme involved in ADP-heptose biosynthesis pathway is essential for lipopolysaccharide biosynthesis and bacterial virulence

**DOI:** 10.1080/21505594.2021.1938449

**Published:** 2021-06-14

**Authors:** Sue-Fen Chiu, Kai-Wen Teng, Po-Chuan Wang, Hsin-Yu Chung, Chun-Jen Wang, Hui-Chun Cheng, Mou-Chieh Kao

**Affiliations:** a Institute of Molecular Medicine, College of Life Science, National Tsing Hua University, Hsinchu, Taiwan; b Department of Gastroenterology, Hsinchu MacKay Memorial Hospital, Hsinchu, Taiwan; c Institute of Bioinformatics and Structural Biology, College of Life Science, National Tsing Hua University, Hsinchu, Taiwan; d Department of Life Science, College of Life Science, National Tsing Hua University, Hsinchu, Taiwan

**Keywords:** *Helicobacter pylori*, D-*glycero*-D-*manno*-heptose-1, 7-bisphosphate phosphatase (GmhB), heptose biosynthesis, lipopolysaccharide, outer membrane vesicle, bacterial pathogenesis, drug target, virulence factor, enzyme kinetics

## Abstract

*Helicobacter pylori* infection is linked to serious gastric-related diseases including gastric cancer. However, current therapies for treating *H. pylori* infection are challenged by the increased antibiotic resistance of *H. pylori*. Therefore, it is in an urgent need to identify novel targets for drug development against *H. pylori* infection. In this study, *HP0860* gene from *H. pylori* predicted to encode a D-*glycero*-D-*manno*-heptose-1,7-bisphosphate phosphatase (GmhB) involved in the synthesis of ADP-L-*glycero*-D-*manno*-heptose for the assembly of lipopolysaccharide (LPS) in the inner core region was cloned and characterized. We reported HP0860 protein is monomeric and functions as a phosphatase by converting D-*glycero*-D-*manno*-heptose-1,7-bisphosphate into D-*glycero*-D-*manno*-heptose-1-phosphate with a preference for the β-anomer over the α-anomer of sugar phosphate substrates. Subsequently, a HP0860 knockout mutant and its complementary mutant were constructed and their phenotypic properties were examined. HP0860 knockout mutant contained both mature and immature forms of LPS and could still induce significant IL-8 secretion after gastric AGS cell infection, suggesting other enzymatic activities in HP0860 knockout mutant might be able to partially compensate for the loss of HP0860 activity. In addition, HP0860 knockout mutant was much more sensitive to antibiotic novobiocin, had decreased adherence abilities, and caused less classic hummingbird phenotype on the infected AGS cells, indicating *H. pylori* lacking HP0860 is less virulent. Furthermore, the disruption of *HP0860* gene altered the sorting of cargo proteins into outer membrane vesicles (OMVs). The above findings confirm the importance of HP0860 in LPS core biosynthesis and shed light on therapeutic intervention against *H. pylori* infection.

## Introduction

*Helicobacter pylori* is a well-recognized pathogen that chronically infects up to half of the world’s population [1–3]. It can colonize the mucosa of human stomach, and has been considered as an important risk factor for the development of peptic ulcer, gastric cancer and mucosa-associated lymphoid tissue lymphoma (MALT) [4–7].

**Figure 1.**
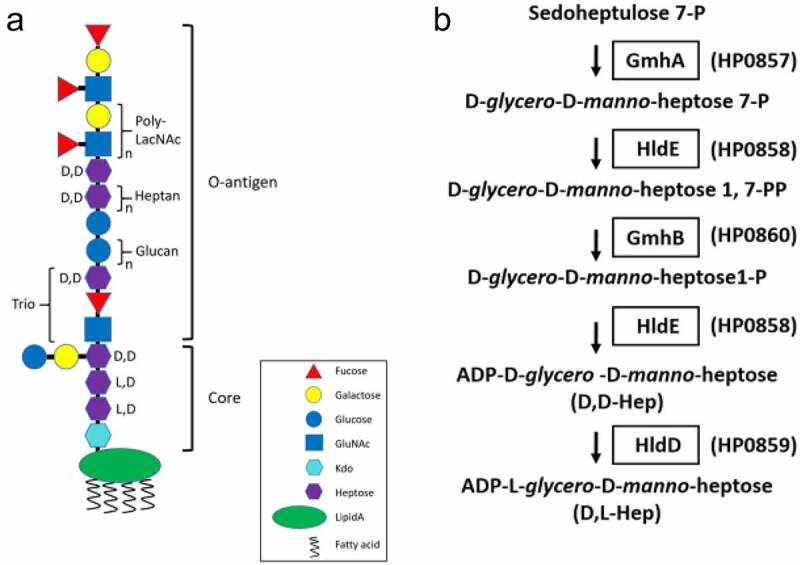
LPS structure and ADP-L-*glycero*-D-*manno*-heptose biosynthetic pathway in *H. pylori. A*, The proposed LPS structure in wild type *H. pylori* 26,695 strain. *B*, The putative biosynthetic pathway of ADP-L-*glycero*-D-*manno*-heptose in *H. pylori*. GmhA, sedoheptulose 7-phosphate isomerase; GmhB, D-α,β-D-heptose 1,7-bisphosphate phosphatase; HldE, bifunctional D-β-D-heptose 7-phosphate kinase/D-β-D-heptose 1-phosphate adenyltransferase; HldD, ADP-D-β-D-heptose epimerase

Lipopolysaccharide (LPS) plays a critical role for the virulence of *H. pylori* infection [8]. LPS is a major component of the outer membrane of Gram negative bacteria, and is generally considered to be toxic with potent immune-modulating and immune-stimulating properties [9,10]. Based on recent research findings, a new LPS structure of *H. pylori* was defined [11,12]. The complete LPS structure, which is called the smooth-form LPS (S-LPS), is composed of three regions including the lipid A, the core oligosaccharide, and the O-antigen in a linear arrangement (Figure 1a). In *H. pylori*, the core oligosaccharide is composed of one 3-*deoxy*-D-*manno*-oct-2-ulsonic acid (Kdo), two connecting L-*glycero*-D-*manno* heptoses (L, D Hep), and one D-*glycero*-D-*manno* heptose (D, D Hep) with a branched disaccharide (galactose and glucose). The core oligosaccharide is linked to a unique linear O-antigen which contains the Lewis X and Lewis Y structure in the terminus to mimic host cell surface antigens and is considered to be the key factor for immune surveillance [13–15].

Heptose (Hep), a special glycan which is only synthesized in prokaryotes, is an important building block of LPS in a broad-spectrum of Gram negative bacteria [16,17]. The D, D Hep and L, D Hep are the main components of the LPS core and the D, D Hep also forms a heptan repeat unit in the O-antigen region. Previous researches have shown that the lack of heptose would result in LPS truncation in many Gram negative bacteria and cause profound effects on bacterial invasion, outer membrane integrity and surface hydrophilicity [18–20]. Recently, there has been a growing number of publications demonstrating that several precursors of heptose can also be a pathogen-associated molecular pattern (PAMP), a molecule derived from pathogens and recognized by pattern recognition receptors (PRRs) of host cells, in addition to being a part of LPS. It has been reported that a heptose precursor, D-*glycero*-D-*manno*-heptose-1,7-biphosphate (HBP) can be recognized by mammalian cells through the ALPK1-TIFA-TRAF6 axis and trigger NF-κB-related innate immune responses during bacterial infections by *Neisseria meningitidis, Neisseria gonorrhoeae, Shigella flexneri* and *Yersinia pseudotuberculosis* [21–24]. Recent studies have also shown that *H. pylori* can inject HBP into mammalian cells through the type IV secretion system [25–27]. Taken together, heptose and its derivatives are considered as the newly discovered virulence factors in Gram negative bacteria and possess great potential to be the targets for the development of new anti-bacterial agents.

To further understand the characteristics of heptose derivatives and their association with *H. pylori* infection, the functions of enzymes involved in the biosynthetic pathway of heptose production need to be fully elucidated. The complete biosynthetic pathway of the ADP-L-*glycero*-D-*manno*-heptose (ADP-L-D-heptose) in *Escherichia coli* has been completely documented [28], and its corresponding pathway in *H. pylori* is shown in Figure 1b. It includes 1) the conversion of D-sedoheptulose-7-phosphate to D-*glycero*-D-*manno*-heptose-7-phosphate by sedoheptulose-7-phosphate isomerase (GmhA); 2) the formation of D-*glycero*-D-*manno*-heptose-1,7-bisphosphate catalyzed by the kinase activity of a bifunctional D-*glycero*-β-D-*manno*-heptose phosphate kinase/D-*glycero*-β-D-*manno*-heptose-1-phosphate adenyltransferase (HldE); 3) the conversion of D-*glycero*-D-*manno*-heptose-1,7-bisphosphate to D-*glycero*-D-*manno*-heptose 1-phosphate by D-*glycero*-α,β-D-*manno*-heptose-1,7-bisphosphate phosphatase (GmhB); 4) the activation of D-*glycero*-D-*manno*-heptose-1-phosphate intermediate to ADP-D-*glycero*-D-*manno*-heptose by the adenyltransferase activity of HldE; and 5) the epimerization of ADP-D-*glycero*-D-*manno*-heptose to form the final product, ADP-L-*glycero*-D-*manno*-heptose by ADP-D-*glycero*-β-D-*manno*-heptose epimerase (HldD). The functions of GmhA (HP0857 protein) and HldD (HP0859 protein) in *H. pylori* have been investigated by our laboratory previously [29,30]. We demonstrated that these two proteins are essential for the biosynthesis of the LPS core in *H. pylori* and have dramatic effects on bacterial survival and virulence. However, what remain lacking are the complete map of heptose biosynthesis and its physiological influence on *H. pylori* infection. In this paper, we aimed to pinpoint another protein, HP0860, possibly involved in the heptose biosynthetic pathway, and analyze the impact of this protein in *H. pylori*’s physiology and virulence.

GmhB is documented to be involved in two glycan synthesis pathways: D-*glycero*-D-*manno*-heptose-1α-GDP for S-layer glycoprotein biosynthesis and L-*glycero*-D-*manno*-heptose−1β-ADP for LPS biosynthesis. In the later pathway, this enzyme is responsible for catalyzing the removal of phosphate group at the C-7 position of D-*glycero*-D-*manno*-heptose-1,7-bisphosphate to produce D-*glycero*-D-*manno*-heptose-1-phosphate [31–33]. The homologues of *HP0860* gene previously referred to as *gmhX* in *N. meningitides* and *gmhB* (formerly *yaeD*) in *E. coli* have been characterized [17,34]. It was also reported that the *gmhB* knockout mutant in *E. coli K-12* strain and *Yersinia pestis* would result in the formation of an altered LPS core, indicating that GmhB protein is required for LPS biosynthesis [34,35]. The three-dimensional (3D) structure of GmhB in *E. coli, Bordetella bronchiseptica* and *Mycobacterium tuberculosis*, and the potential mechanisms of its catalytic reaction have been unraveled [31–33,36].

In this study, the biological functions of HP0860 protein in *H. pylori* 26,695 strain were investigated. We cloned the *HP0860* gene, characterized the biochemical and biophysical properties of HP0860 protein and conducted the enzyme kinetic studies for its phosphatase activity *in vitro*. In addition, we also constructed a HP0860 knockout mutant and its corresponding complementary mutant, and examined the effects of *HP0860* mutations on the phenotype, survival, and virulence of *H. pylori*. The experimental works presented here provide one of the first investigations to detailedly explore the function and effect of HP0860 protein in *H. pylori* with the hope that the acquired knowledge will benefit greatly to combat *H. pylori* infection.

## Materials and methods

### Bacterial strains, plasmids and cell culture

Bacterial strains and plasmids used are listed in Table 1. *H. pylori* 26,695 (ATCC700392) was grown in Brucella broth with 10% fetal bovine serum (Gibco; 16,000,044) and 1% IsoVitalex (Fisher Scientific; BD 211,876) and cultivated for 48 hours under a microaerophilic condition (5% O_2_, 10% CO_2_ and 85% N_2_) at 37°C with shaking. *E. coli* cells were propagated in Luria-Bertani broth at 37°C and selectively supplemented with the following antibiotics: 50 μg/mL kanamycin (MDbio; K001), 100 μg/mL ampicillin (USB; J11259), and 10 μg/mL chloramphenicol (Sigma-Aldrich; C0378). Gastric AGS cells (ATCC CRL-1739) were grown in Ham’s F-12 medium (Sigma-Aldrich; N6760) containing 10% fetal bovine serum. Cells were incubated under a humidified atmosphere of 95% air and 5% CO_2_ at 37°C.

**Table 1.** Bacterial strains and plasmids used in this study

Strain/plasmid		Characteristics & marker^a^	Source
**Strain**	***E. coli***		
	Top10	Host for construction of pGEM-T-HP0860 clones	Invitrogen, Carlsbad, USA
	DH5α	Host for construction of pGEM-T-HP0860^d^:: Cm^r^ clones	Invitrogen, Carlsbad, USA
	BL21 (DE3)	Host for expression of recombinant HP0860 protein	Novagen, Darmstadt, Germany
	***H. pylori***		
	26,695	*H. pylori* whole-genome sequencing strain, isolated from the stomach of a patient with gastritis	ATCC 700,392
	HP0860 KO	*H. pylori* 26,695 strain with a chloramphenicol resistance cassette in *HP0860*; Cm^r^	this study
	HP0860 Com	HP0860 KO with *HP0860* insertion in *HP0954* (*RdxA*); Met^r^, Cm^r^	this study
**Plasmid**	pGEM-T	T-A cloning vector; Amp^r^	Promega, Madison, USA
	pGEM-T-HP0860	pGEM-T with HP0860 DNA fragment; Amp^r^	this study
	pGEM-T-HP0860^d^:: Cm ^r^	pGEM-T with *HP0860* interrupted with a chloramphenicol resistance cassette; Cm^r^	this study
	pGEM-T-RdxA_L_-P_HP1563_- HP0860-T7_ter_-RdxA_R_	pGEM-T with *HP0860* insertion in *HP0954* (*RdxA*); Met^r^, Cm^r^	this study
	pET28a	Protein expression vector; Kan^r^	Novagen, Darmstadt, Germany
	pET28a-HP0860	Protein expression vector for recombinant HP0860 protein, Kan^r^	this study

***^a^***Amp^r^, ampicillin resistant; Kan^r^, kanamycin resistant; Cm^r^, chloramphenicol resistant; Met^r^, metronidazole resistant.

### Molecular cloning of *HP0860* gene

The targeted *HP0860* gene was amplified with a primer set (Hp0860F and Hp0860R) listed in Table 2 by using *H. pylori* 26,695 genomic DNA as the template. The amplification was conducted with *Pfu* DNA polymerase (Promega; M7741). The obtained PCR product was ligated into a pGEM-T vector (Promega; A3600) and the resulting vector (pGEM-T-HP0860) was transformed into *E. coli* Top10 competent cells. The plasmid with a correct *HP0860* gene fragment was digested with restriction enzymes NdeI and XhoI and the released fragment was then subcloned into an expression vector pET28a (Novagen; 69,864), and the obtained plasmid (pET28a-HP0860) was then transformed into competent *E. coli* BL21 (DE3) cells (Novagen; 69,450). The resulting cells were spread on an agar plate with kanamycin for selection.

**Table 2.** Sequences of the primers used in this study

Primer	Restriction site	Sequence (5ʹ→3ʹ)^a^
Hp0860F	NdeI	GCATTCATATGAACACTAACAAAGCCC
Hp0860R	XhoI	TTACTCTCGAGCGTGTTACTGCGG
KO0860F1	NdeI	CTGCATATGCCGTTTCTTACAGGAAATTT
KO0860R1	BamHI	CTGGATCCTTGGCTTCCTGCAAGCGCAA
KO0860F2	BamHI	AATTAGGGATCCGATCAACCGAGGCTAT
KO0860R2	XhoI	CTGCTCGAGTTAGGGTGGTTCTCTTGAAA
Com0860F1	NcoI	CATGCCATGGAACGATCCTTTTTGTATGATTTAT
Com0860R1	-	AGGGCTTTGTTAGTGTTCATATCGTAACTCCTTAAG
Com0860F2	-	ATGAACACTAACAAAGCCCTTTTTTTGGACA
Com0860R2	KpnI	CTGGGTACCTTATTTGATTAGATCTATCATCTC
Rdx _L_F	ApaI	GGGCCCGCATTCGTGGGATGAGCTA
Rdx _L_R	NcoI	CCATGGCTTGCAAGAATGGCGCTCG
Rdx _R_F	PstI	ATGCCTGCAGGTGGCAGAAGCGAGTCA
Rdx _R_R	SacI	GTAGAGCTCGCTCAATCTGACAAC CCCAC
T7terF	SacI	TAATACGAGCTCACTATAGG
T7terR	NotI	AATCGGCGGCCGCAAAAAACCCCTCAAGACC

**^a^**The sequences of restriction enzyme cutting sites are underlined.

### HP0860 protein expression and purification

The transformed BL21 (DE3)/pET28a-HP0860 cells were grown in 2× YT medium containing 50 μg/mL kanamycin with shaking. When OD_600_ reached 0.6–0.8, the transformed cells were induced with 1 mM isopropyl-β-D-thiogalactoside (IPTG; Sigma-Aldrich; I6758) for 4 hours at 37°C. The IPTG-induced cells were harvested by centrifugation (4000×g for 20 minutes at 4°C), and then the cell pellet was suspended in 30 mL of homogenization buffer (20 mM Tris-HCl (pH 7.4), 500 mM NaCl, 20 mM imidazole) and passed through a high-pressure homogenizer (EmulsiFlex-C5; AVESTIN; Canada) to break cells. The obtained lysate was centrifuged (11,000×g, 20 minutes at 4°C), and the resulting supernatant was applied for affinity purification with IMAC Sepharose 6 FastFlow resins (GE Healthcare; 17–0921-08) and 100 mM CoCl_2_ as the immobilized metal ion according to manufacturer’s recommendation. After thoroughly washing, the protein was eluted with a buffer containing 250 mM imidazole. The desired eluted fractions were combined and concentrated by using an Amicon Ultra 15 concentrator (Millipore; Z717185) to obtain recombinant HP0860 protein.

### Construction of HP0860 knockout mutant and its corresponding complementary mutant

HP0860 knockout mutant was constructed using a strategy based on gene splicing by overlap extension (SOEing) [37–39]. The upstream (270 bp) and downstream (259 bp) regions of *HP0860* gene were amplified by PCR from *H. pylori* 26,695 genomic DNA using primer pairs KO0860F1-KO0860R1 and KO0860F2-KO0860R2, respectively (Table 2). The two PCR products generated were then joined via SOEing PCR with primers KO0860F2 and KO0860R1 which generated BamHI sites for subsequent insertion of the antibiotic resistance cassette to disrupt *HP0860* gene. The amplified 500 bp *HP0860* deletion fragment was digested with BamHI, gel purified, and cloned into the pGEM-T vector to create the pGEM-T-HP0860^d^ plasmid. The resulting plasmid was then transformed into *E. coli*, and the transformed bacteria were screened for an insert via PCR and the correct construct was further verified by restriction digestion. Later, a chloramphenicol resistance cassette (*Cm^r^*), which was pre-digested with BamHI, was inserted into the pGEM-T-HP0860^d^ plasmid that has been digested with BamHI and treated with shrimp alkaline phosphatase. The resulting plasmid, pGEM-T- HP0860^d^:: *Cm^r^*, was then introduced onto the chromosome of wild type *H. pylori* 26,695 strain by nature transformation, and the resistant colonies were selected on sheep blood agar plates containing 34 μg/mL of chloramphenicol. The successful insertion of the resistance cassette was confirmed by PCR analysis using primers KO0860F1 and KO0860R2.

To complement the aforementioned HP0860 knockout mutation, the strategy of chromosomal complementation was adopted using the *rdxA* (*HP0954*) system [40]. When the *rdxA* (*HP0954*) locus was disrupted by an insertion of *HP0860* gene, it would confer metronidazole (Mtz) resistance to bacteria. The procedure for construction and selection of HP0860 complementary mutant was conducted as described by Croxen et al. with some modification [41]. First, the pGEM-T-RdxA_L_-T7_ter_-RdxA_R_ plasmid was constructed as follows. Approximately 580 bp flanking regions both for upstream (RdxA_L_) and downstream (RdxA_R_) of *rdxA* were amplified by PCR using primer pairs RdxA_L_F-RdxA_L_R and RdxA_R_F-RdxA_R_R, respectively. The resulting fragments were then digested with ApaI/NcoI and PstI/SacI, respectively, and sequentially cloned into the pGEM-T to obtain the pGEM-T-RdxA_L_-RdxA_R_ plasmid. Next, a T7 bacteriophage transcriptional terminator was amplified with primers T7terF and T7terR, digested with XhoI and NotI, and inserted into the pGEM-T-RdxA_L_-RdxA_R_ plasmid to generate the pGEM-T-RdxA_L_-T7_ter_-RdxA_R_ plasmid. Then, the promoter region of *HP1563* gene (240 bp) and the ORF of *HP0860* gene (522 bp) were amplified from *H. pylori* 26,695 genomic DNA by PCR using primers pairs Com0860F1-Com0860R1 and Com0860F2-Com0860R2, respectively (Table 2). The above two PCR products generated were then joined via SOEing PCR with primers Com0860F1 and Com0860R2. The resulting 762 bp product was then digested with NcoI/KpnI and ligated into the pGEM-T-RdxA_L_-T7_ter_-RdxA_R_ plasmid precut with the same restriction enzymes to create pGEM-T-RdxA_L_-P*_HP1563_*-HP0860-T7_ter_-RdxA_R_ plasmid. The resulting complementary plasmid was then transformed into HP0860 knockout mutant by nature transformation. Through homologous recombination, resistant colonies were selected on sheep agar plates containing 34 μg/mL of chloramphenicol and 8 μg/mL of metronidazole. The correct insertion of *HP0860* gene in the *rdxA* locus was confirmed by PCR analysis.

### Determination of molecular weight and oligomeric state of HP0860 protein

The molecular weight of a single subunit of HP0860 protein was measured by matrix-assisted laser desorption ionization-time of flight mass spectrometry (MALDI-TOF MS). The purified HP0860 protein was desalted using an Amicon concentrator (with 10 kDa cutoffs) before being analyzed. Further, the native molecular weight of HP0860 protein was characterized both by analytical ultracentrifugation using the Beckman Optima™ XL-A Analytical Ultracentrifuge (Beckman Coulter; USA) and by gel filtration using a HiLoad 16/600 Superdex 200 pg column (GE Healthcare; 28–9893-35) and AKTA FPLC instrument at 4°C equilibrated with a buffer contained 20 mM HEPES (pH 8.0), 10 mM MgCl_2_ and 10 mM KCl.

### Circular dichroism analysis of the secondary structure of HP0860 protein

The circular dichroism spectra of HP0860 protein were recorded using an Aviv 62DS spectropolarimeter (Aviv Associates; USA). Samples were prepared at a concentration of 30 μM in a buffer containing 5 mM HEPES (pH 8.0) and were loaded into a quartz cuvette with 1 mm path length (Hellma Analytics; Germany). The CD spectra were plotted as mean residue molar ellipticity (degrees×cm^2^×dmol^−1^) vs. wavelength (nm) from 190 to 260 nm, and the content of the secondary structure of HP0860 protein was estimated from the CD spectra using the K2D3 program (http://www.ogic.ca/projects/k2d3/).

### Kinetic analysis of HP0860 protein

The enzymatic activity of HP0860 (GmhB), which is predicted to catalyze the conversion of D-*glycero*-D-*manno*-heptose-1,7-bisphosphate into D-*glycero*-D-*manno*-heptose-1-phosphate by dephosphorylation in the ADP-L-D-heptose biosynthesis pathway, was measured spectrophotometrically by using the Malachite Green Phosphate Assay (Kits Bioassay Systems; POMG-25 H) to monitor phosphate release. At first, a series of diluted standards from the stock solution provided in the Malachite Green Phosphate kit were prepared and used to generate a standard curve. Subsequently, the assays were executed in a buffer containing 20 mM HEPES (pH 8.0), 10 mM MgCl_2_, 10 mM KCl and 0.0024 nmol recombinant HP0860 to a final volume of 90 μL. The reactions were initiated by the addition of 10μL tested substrate with a final concentration varied from 0 to 2 mM. After incubation at 25${\;}^{\circ }C$ for 30 minutes, the reaction was stopped by the addition of 10 μL of 5% trichloroacetic acid for 1 minute at 4${\;}^{o}C$. Then, 80 μL of the reaction mixture with phosphate released was then transferred to a 96-well microplate and added with 20 μL of a mixture of ammonium molybdate and malachite green hydrochloride in 4 M HCl provided by Malachite Green Phosphate Assay kit. The assays were then incubated for color development at 25${\;}^{o}C$ for 30 minutes. The absorbance was measured at 620 nm wavelength on a Wallac 1420 Victor plate reader and all reactions were repeated in triplicate. The background level of phosphate release was determined by the corresponding control reaction without the addition of HP0860 protein and deducted from each of the obtained points. The rate of reaction (amount of phosphate released/minute) was calculated and the data of initial reaction velocity were fitted to the Michaelis–Menten equation V_0_ = V_max_[S]/(K_m_+[S]), where V_0_ is the initial velocity, V_max_ is the maximal velocity, [S] is the substrate concentration, and K_m_ is the Michaelis–Menten constant for the substrate used, with Prism (GraphPad Software; USA). The turnover number k_cat_ was calculated from V_max_ and [E] according to the equation k_cat_ = V_max_/[E], where [E] is the enzyme concentration. The assay substrates used in this study include D-*glycero*-D-*manno*-heptose-1α,7-bisphosphate and D-*glycero*-D-*manno*-heptose-1β,7-bisphosphate, both were kindly provided by Dr Karen N. Allen (Boston University, Boston, USA).

### Growth curve analysis of *H. pylori*

Wild type *H. pylori* 26,695 strain, HP0860 knockout mutant and HP0860 knockout complementary mutant were grown in Brucella broth including 10% fetal bovine serine and 1% IsoVitale X under microaerophilic conditions at 37°C for 16 hours. The overnight bacterial suspension was later diluted to an OD_600_ of 0.01 and incubated with shaking at 140 r.p.m. under microaerophilic conditions for up to 72 hours. The absorbance was measured at 600 nm wavelength by spectrophotometer at various time points, and the experiment was repeated in triplicate.

### Novobiocin sensitivity assay

Wild type *H. pylori* 26,695 strain, HP0860 knockout mutant and HP0860 knockout complementary mutant were grown in Brucella broth for 48 hours at 37${\;}^{o}C$ and later diluted to $OD_{600}$ = 0.6. The resulting cell suspension was treated with different concentration of novobiocin (0, 20, 40, 60, 80, 100μg/mL). 1 mL of the above cell suspension was placed in 24-well plates in triplicate and incubated under microaerophilic conditions at 37${\;}^{o}C$ with 140 r.p.m. shaking. The $OD_{600}$ value of bacterial culture was then measured after 72 hours of incubation. The survival rate was defined as followed: survival rate (%) = $OD_{600}$ of bacterial growth with novobiocin treatment/$OD_{600}$ of bacterial control without novobiocin treatment.

### Isolation and analysis of *H. pylori* OMVs

OMVs were isolated from culture supernatants using a method described by Wai et al. with some modifications [42]. Briefly, different strains of *H. pylori* suspension harvested at log (OD_600_ ≅ 1.0) or stationary phase (OD_600_ ≅ 2.0) during growth in a final volume of 25 mL were centrifuged twice (12,000 ×g for 10 minutes, 4${\;}^{o}C$). The culture supernatants were filtered (with 0.45 μm membranes) then ultra-centrifuged (30,000 ×g for 2 hours, 4${\;}^{o}C$) to recover OMVs. The pellets containing OMVs were washed twice with phosphate-buffered saline (PBS) and finally re-suspended in 0.02 M Tris-HCl (pH 8.0) for further study. For the investigation of protein contents in OMVs, purified OMVs were analyzed by 15% SDS-PAGE with Coomassie blue staining and by immune-blotting analysis. For the analysis of LPS in OMVs, LPS obtained from OMVs were treated with protease K and extracted by the phenol-water method, followed by the analysis of LPS profile with a similar manner as described below.

### SDS–PAGE and immuno-blotting analysis

Protein samples were separated on 10% or 15% gels by sodium dodecyl sulfate–polyacrylamide gel electrophoresis (SDS–PAGE) and stained with 0.25% Coomassie Brilliant Blue R250 (Sigma-Aldrich; 20,278) reagent or transferred to 0.45 μm polyvinylidene fluoride membranes (Pall; BSP0861) for immuno-blotting analysis. The membranes were incubated at 4°C overnight with one of the following primary antibodies: mouse-anti-His antibody (1:200; Santa Cruz biotechnology; sc-8036), mouse-anti-Lewis Y monoclonal antibody (1:1000, Santa Cruz Biotechnology; sc-59,472), rabbit-anti-CagA monoclonal antibody (1:2000; Santa Cruz Biotechnology; sc-25,766), goat-anti-UreA monoclonal antibody (1:3000; Santa Cruz Biotechnology; sc-21,016), and visualized by LI-Cor Odyssey® Infrared Imaging System.

### LPS profile analysis

LPS from different strains of *H. pylori* was prepared by a hot phenol-water method [43]. The extracted LPS was separated by 15% SDS-PAGE and visualized by silver staining as described previously [44,45], or by immuno-blotting analysis using monoclonal mouse-anti-Lewis Y antibody.

### Infection and adhesion assay

AGS cells were seeded at 2 × 10^5^ cells/well in 24-well plates containing Ham’s F-12 medium supplemented with 10% FBS under the standard cell culture conditions and allowed to adhere overnight. When AGS cells reached approximately 80% confluency, the medium was aspirated and the cells were washed twice each with 1 mL of PBS. Different strains of *H. pylori* suspension harvested at log phase of growth (OD_600_ ≌ 1.0) were added to AGS cells at a multiplicity of infection (MOI) of 100. After 4 hours of co-culture, the morphological changes of these cells were recorded by using a IX71 research inverted system microscope (Olympus; Japan) with 20 × magnification. For adhesion assay, bacteria were co-cultured with AGS cells for 4 or 6 hours. The non-adhered bacteria were removed by PBS buffer wash and then the cells were lysed by treating with 0.5% saponin-containing PBS buffer for 5 minutes of incubation. The obtained lysate was serially diluted and spread on sheep blood agar plates. The adhered bacteria were measured by the viable plate counting method after 72 to 96 hours of incubation.

### Determination of IL-8 secretion

AGS cells were cultivated and synchronized to 2 × 10^5^ cells at each well and then infected by various strains of *H. pylori* at 100 MOI for 4 hours. The condition medium was collected after the co-incubation and then subjected to determine the secretion of IL-8 by a commercial human IL-8 ELISA kit (Human IL-8 ELISA Ready-SET-Go!; Affymetrix ebioscience; 88–8086) according to the supplier’s protocol.

### Statistical analysis

Unless otherwise noted, each experiment was conducted at least three independent times. All values are given as the mean ± S.D. Statistical analysis was performed by using the two-tailed student’s t test. The significance was labeled by *<0.05, **<0.01 and ***<0.001. A value of p < 0.05 (*) was considered statistically significant.

## Results

### HP0860 protein in wild type *H. pylori* 26,695 strain is a putative HBP phosphatase

**Figure 2.**
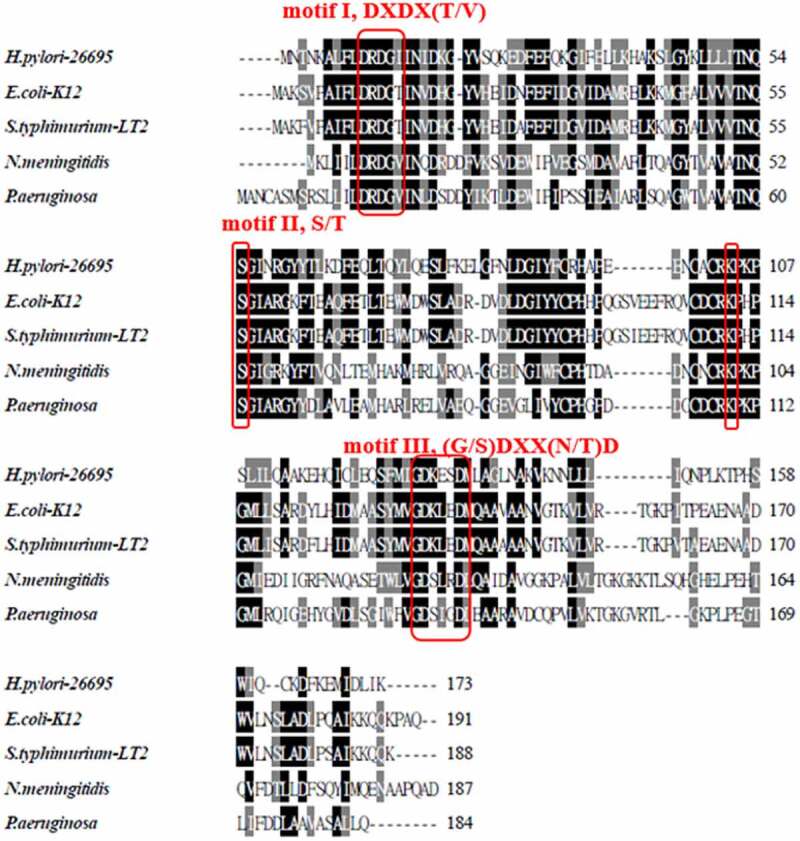
Multiple sequence alignment of HP0860 protein. Sequence alignment of HP0860 protein in *H. pylori* 26,695 strain with GmhB proteins in other Gram negative bacteria. The result of alignment was generated by Clustal W2 (http://www.ebi.ac.uk/Tools/clustalw2/) and displayed by BOXSHADE server (http://www.ch.embnet.org/software/BOX_form.html). Sequence sources and their Swiss-Prot accession numbers are as follows: *Helicobacter pylori* 26,695 [O25531], *Escherichia coli* K-12 [P63228], *Salmonella typhimurium* LT2 [Q8ZRM8], *Neisseria meningitidis* Z2491 [Q9JWE9] and *Pseudomonas aeruginosa* PAO1 [Q9I7C0]

*HP0860* gene was suggested to encode a protein with 173 amino acid residues. The amino acid sequences of HP0860 and GmhB from different species of Gram negative bacteria were aligned using ClustalW2 program (Figure 2), and the result is summarized in Table 3. HP0860 protein of wild type *H. pylori* 26,695 strain displays a high degree of sequence homology with GmhB of other Gram negative bacteria (40.6% identity for *Escherichia coli* K-12, 30.4% identity for *Salmonella typhimurium* LT2, 31.6% identity for *Neisseria meningitidis* Z2491 and 35.1% identity for *Pseudomonas aeruginosa* PAO1). According to the sequence annotation, GmhB has a conserved haloacid dehalogenase-like (HAD) domain which is possessed by the members of HAD protein superfamily. The HAD domain contains three conserved sequence motifs: DXDX(T/V), S/T and (G/S)DXX(N/T)D [46]. Alignment of HP0860 protein showed that it has the same sequence motifs as GmhB in other bacteria (Figure 2). These results indicated that HP0860 protein could be a putative GmhB, which is responsible for catalyzing the conversion of D-*glycero*-D-*manno*-heptose-1,7-bisphosphate into D-*glycero*-D-*manno*-heptose-1-phosphate in the third step of ADP-L-D-heptose biosynthetic pathway in *H. pylori*.

**Table 3.** Sequence comparison of *H. pylori* HP0860 protein with homologues from other Gram negative bacteria

Species	UniProt AC	% Identity^a^	% Similarity^a^	Length
***Helicobacter pylori* 26,695**	O25531	100%	100%	173
***Escherichia coli* K-12 -MG1655**	P63228	40.6%	60.6%	191
***Salmonella typhimurium* LT2**	Q8ZRM8	30.4%	51.1%	188
***Neisseria meningitidis* Z2491**	Q9JWE9	31.6%	55.6%	187
***Pseudomonas aeruginosa* PAO1**	Q9I7C0	35.1%	53.4%	178

^a^Data were obtained by the Comprehensive Microbial Resource, CMR website (http://cmr.jcvi.org/cgi-bin/CMR/CmrHomePage.cgi).

### Biophysical and structural characterization of HP0860 protein

**Figure 3.**
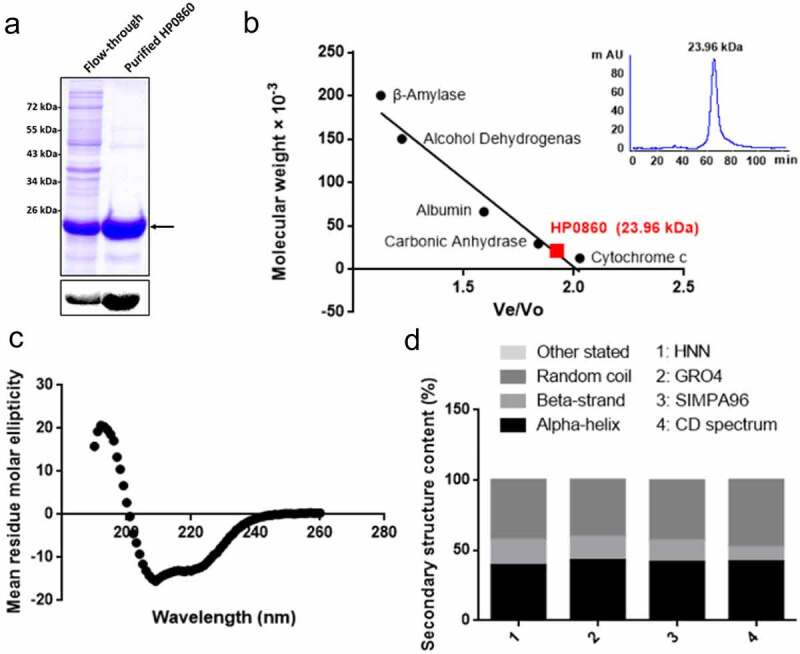
Purification and characterization of the recombinant HP0860 protein. *A*, Purification of recombinant HP0860 protein. Protein was collected and concentrated from the eluted fractions and analyzed by 15% SDS–PAGE with Coomassie blue staining and immuno-blotting analysis using anti-His antibody. *B*, The native molecular weight of HP0860 was determined by gel filtration. The molecular weight determination of HP0860 was made by comparing the ratio of Ve/Vo for HP0860 to the Ve/Vo of protein standards of known molecular weights (Ve: elution volume; Vo: void volume for blue dextran 2000). *C*, The secondary structure of HP0860 was analyzed by circular dichroism (CD) spectra, which were measured from 190 to 260 nm at 25${\;}^{o}C$. *D*, The content of the secondary structure of HP0860 protein. The secondary structural prediction based on HP0860 amino acid sequence was made by using the Web-based algorithms HNN, GRO4 and SIMPA96 available at the Network Protein Sequence @nalysis (NPS@) website (http://npsa-pbil.ibcp.fr)

To investigate the biophysical and biochemical properties of HP0860 protein, recombinant HP0860 protein with a 6×His tag was expressed and purified (Figure 3a). The molecular size and the oligomeric state of purified HP0860 protein were examined by Matrix-assisted laser desorption ionization time of flight mass spectrometry (MALDI-TOF MS), gel filtration chromatography and sedimentation velocity ultracentrifugation. Results from MALDI–TOF MS spectrometry revealed that the purified HP0860 protein had a single subunit molecular weight with a major peak at m/z 22.09 kDa (Supplementary Fig. 1S) which agreed very well with a molecular weight of 22 kDa as analyzed by SDS-PAGE (Figure 3a). The native molecular weight of HP0860 protein estimated by gel-filtration chromatography under non-denaturing conditions showed a protein peak corresponding to a molecular weight of 23.96 ± 0.10 kDa (Figure 3b). The result from analytical ultracentrifugation indicated that HP0860 protein had a native molecular weight of 27.02 ± 0.20 kDa (Supplementary Fig. 2S). Altogether, these results indicated that the oligomerization state of the HP0860 protein in solution appeared to be monomeric with a molecular weight about 22 kDa.

The secondary structure of purified HP0860 protein was analyzed by circular dichroism (CD) spectroscopy. The obtained CD spectrum exhibited a maximum and a minimum around 198 and 207 nm, respectively, which was an indication of α-helical structure (Figures 3c and 3d). Further, the contents of the secondary structure of HP0860 protein were estimated from the CD spectra using the K2D3 program, revealing 41.88% α-helix and 10.11% β-strand secondary structure contents in HP0860 protein. The prediction of secondary structure based on HP0860 amino acid sequence was made by using the Web-based algorithms HNN, GRO4 and SIMPA96 (http://npsa-prabi.ibcp.fr). Based on these analyses, it was estimated that the α-helix and β-strand contents of HP0860 ranged from 39.31% to 42.77% and from 15.52% to 17.92%, respectively (Figure 3d). The results of the predicted secondary structural elements for *H. pylori* HP0860 protein were similar to those obtained from direct CD analysis in this study. On top of that, the structural modeling in *E. coli* GmhB suggested that it indeed possesses a core α/β domain, and a recent structural analysis in *M. tuberculosis* GmhB also indicated that it contains 38% α-helix and 15% β-strand domains [36], which are all in agreement with our CD data. Therefore, it is suggested the *H. pylori* HP0860 protein has a similar secondary structure ratio to other GmhB homologues.

### Determination of enzymatic activity and substrate specificity of HP0860 protein

The catalytic activity of HP0860 (GmhB), expected for the conversion of D-*glycero*-D-*manno*-heptose-1,7-bisphosphate into D-*glycero-Dmanno*-heptose-1-phosphate by dephosphorylation in the ADP-L-D-heptose biosynthesis pathway, was analyzed by using the Malachite Green Phosphate Assay to monitor phosphate releasing. The purified recombinant HP0860 was incubated with increasing concentrations of D-*glycero*-D-*manno*-heptose-1α,7-bisphosphate or D-*glycero*-D-*manno*-heptose-1β,7-bisphosphate, and the rate of reaction (amount of phosphate formation/min) was calculated and the obtained initial reaction velocity was fitted with the Prism software to determine the kinetic parameters. As shown in Table 4, the results revealed that the K_m_ values of HP0860 for D-*glycero*-D-*manno*-heptose-1β,7-bisphosphate and D-*glycero*-D-*manno*-heptose-1α,7-bisphosphate were 0.21 mM and 0.30 mM, respectively (0.7 fold, p = 0.17), suggesting the affinity of HP0860 toward the β-anomer was only slightly higher than that of the α-anomer of sugar phosphate substrates but without statistical significance. However, the catalytic efficiency (measured as k_cat_/K_m_) of recombinant HP0860 for the hydrolysis of D-*glycero*-D-*manno*-heptose-1β,7-bisphosphate was higher than that for the hydrolysis of D-*glycero*-D-*manno*-heptose-1α,7-bisphosphate (10.38 mM^−1^s^−1^versus 3.57 mM^−1^s^−1^), indicating HP0860 has a substrate preference for the β-anomer of its physiological substrate D-*glycero*-D-*manno*-heptose-1,7-bisphosphate. The above finding suggested that HP0860 possesses the phosphatase activity and is capable of catalyzing the conversion of D-*glycero*-D-*manno*-heptose-1,7-bisphosphate into D-*glycero*-D-*manno*-heptose-1-phosphate *in vitro*.

**Table 4.** Steady-state kinetic constants for *H. pylori* GmhB-catalyzed hydrolysis of phosphate esters in 20 mM HEPES (pH 8.0) containing 10 mM MgCl_2_ and 10 mM KCl

Substrate	K_cat_ (s^−1^)	K_m_ (mM)	K_cat_/K_m_ (mM^−1^ s^−1^)
D -*glycero*- D -*manno*-heptose 1α,7-bisphosphate	1.07±0.10	0.30±0.08	3.57
D -*glycero*- D -*manno*-heptose 1β,7-bisphosphate	2.18±0.17	0.21±0.05	10.38

### *HP0860* gene disruption affects *H. pylori* growth and antibiotic novobiocin resistance

To understand the role of HP0860 in *H. pylori*, HP0860 knockout mutant and its corresponding complementary mutant of wild type *H. pylori* 26,695 strain were constructed. *HP0860* gene was disrupted by inserting a chloramphenicol resistance cassette ($Cm^{r}$) into the chromosomal DNA to generate HP0860 knockout mutant, and the resulting mutant was complemented by inserting a DNA fragment containing the promoter region of *HP1563* gene and the ORF of *HP0860* gene into the *rdxA* gene sequence region to generate HP0860 knockout complementary mutant [40]. The correct constructions of these mutants were verified by PCR amplification (Supplementary Fig. 3S).

**Figure 4.**
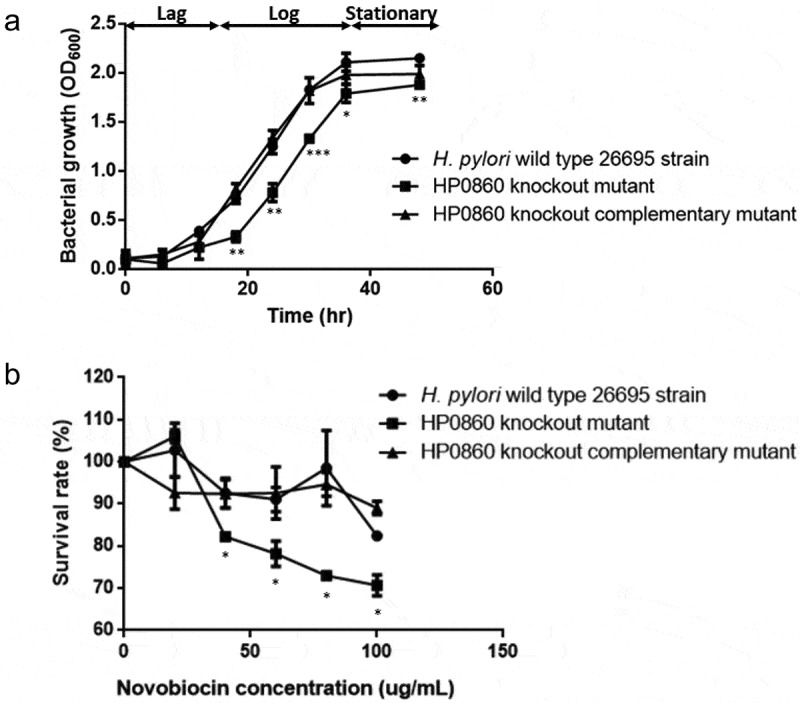
Effects of HP0860 mutations on *H. pylori* growth and antibiotic novobiocin resistance. *A*, The growth curves of different *H. pylori* strains including wild type *H. pylori* strain, HP0860 knockout mutant and HP0860 knockout complementary mutant. The data were recorded by spectrophotometer at $OD_{600}$ at different time points. *B*, Novobiocin sensitivity of different *H. pylori* strains. The effect of HP0860 mutations on novobiocin resistance of *H. pylori* was evaluated by the survival rate which was measured by the ratio of $OD_{600}$ of bacteria incubated with various concentrations of novobiocin and that of the control group without the treatment of antibiotic. Error bars indicated ±SD (n = 3); statistical significance was determined by student’s T test (*p < 0.05, **p < 0.01, ***p < 0.001)

To evaluate the importance of HP0860 in *H. pylori* growth, the growth curves of wild type *H. pylori* strain, HP0860 knockout mutant and HP0860 knockout complementary mutant were investigated (Figure 4a). Wild type *H. pylori* strain and its HP0860 knockout complementary mutant were in the log phase after 12 hours of growth, which is faster than the time required for HP0860 knockout mutant. In addition, HP0860 knockout mutant had a retarded growth in both the log phase (12–36 hours) and the stationary phase (36–48 hours) compared to wild type *H. pylori* strain. The OD_600_ value of HP0860 knockout mutant was less than that of wild type *H. pylori* strain by 54.2%, 37.6% and 27.4% at 18, 24 and 30 hours with significant difference, respectively. All tested strains reached their maximal culture densities during 36 to 48 hours of growth; likewise, the growth of HP0860 knockout mutant was still lower than that of wild type *H. pylori* strain in the stationary phase with statistical significance. (The OD_600_ value of HP0860 knockout mutant was less than that of wild type *H. pylori* strain by 15.2% and 12.6% at 36 and 48 hours, respectively.) However, the analysis from the semi-log plot of the log phase growth revealed that the log phase growth rates were not significantly different among the strains tested (Supplementary Fig. 4S). On the whole, HP0860 knockout mutant seems not only to have an extended lag phase growth but also have a shorter log phase growth relative to wild type *H. pylori* strain and HP0860 knockout complementary mutant, indicating the deficiency of HP0860 protein had an influence on the growth of *H. pylori*.

Novobiocin sensitivity assay was conducted to investigate the effect of HP0860 protein loss on the resistance of *H. pylori* to hydrophobic antibiotics. As shown in Figure 4b, while treated with 100 μg/mL of novobiocin, wild type *H. pylori* strain still had more than 82% survival rate. In contrast, disruption of *HP0860* gene significantly reduced the survival rate of HP0860 knockout mutant even exposed to 40 μg/mL of novobiocin. Nevertheless, the complementation of *HP0860* gene restored the ability of novobiocin resistance in *H. pylori*. These results suggested HP0860 protein is required for maintaining membrane integrity and may contribute to the biosynthesis of LPS structure which works as a protective barrier of *H. pylori* against toxic hydrophobic molecules.

### HP0860 protein is critical for LPS expression

**Figure 5.**
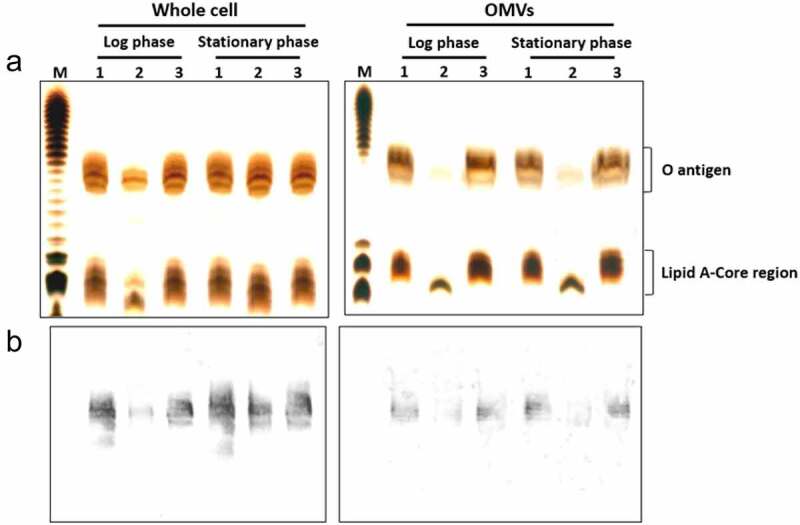
Effects of HP0860 mutations on LPS presentation. Different strains of *H. pylori* were harvested at log or stationary phase during growth and adjusted to the same OD_600_ values. LPS purified from whole cells or OMVs was separated by 15% SDS-PAGE and analyzed by silver staining (*a*) or immuno-blotting using anti-Lewis Y monoclonal antibody (*b*). The relative positions of O-antigen and Lipid A-core region were indicated by braces. M: *E. coli* O111:B4 LPS marker; 1: wild type *H. pylori* strain; 2: HP0860 knockout mutant; 3: HP0860 knockout complementary mutant

The effect of HP0860 knockout mutation on LPS production on *H. pylori* was determined by silver staining and immuno-blotting analysis (Figure 5). Different strains of *H. pylori* were collected at the log and stationary phase of growth and the LPS profiles of different strains of *H. pylori* were analyzed as described in the Method section. The result of silver-stained gel showed a dramatic loss of the O-antigen band pattern and a slightly faster mobility in the LPS core-lipid A region in HP0860 knockout mutant compared to wild type *H. pylori* strain and HP0860 knockout complementary mutant at log phase of *H. pylori* growth (Figure 5a, left panel). However, when grown to stationary phase, HP0860 knockout mutant showed an LPS pattern similar but not identical to that of wild-type *H. pylori* strain and HP0860 knockout complementary mutant. By immune-blotting analysis using O-antigen-specific anti-Lewis Y antibody, the amount of O-antigen at log phase of growth was much less in HP0860 knockout mutant than in wild type *H. pylori* strain, whereas HP0860 knockout mutant produced a similar amount of O-antigen as that of wild type *H. pylori* strain at stationary phase (Figure 5c, left panel). In this respect, we speculated that other protein activities presented in HP0860 knockout mutant might be able to partially compensate for the loss of HP0860 activity for the synthesis of LPS core oligosaccharide. In addition, *H. pylori* has been reported to be capable of producing outer membrane vesicles (OMVs) which contained many of the surface components of *H. pylori* such as LPS [47]. Thus, LPS from OMVs of these strains was also isolated both at log phase and stationary phase of *H. pylori* growth and analyzed. Surprisingly, as shown in right panel of Figure 5a, OMVs obtained from HP0860 knockout mutant exhibited a severely truncated structure of LPS which not only lacked the O-antigen and the major part of the core structure, but also caused the migration of LPS core slightly down-shifted compared to that of wild type *H. pylori* strain and HP0860 knockout complementary mutant both at log phase and stationary phase of growth. Accordingly, the result of immune-blotting analysis also indicated that OMVs from HP0860 knockout mutant had lost most of the Lewis Y determinants in O-antigen both at log phase and stationary phase of growth (Figure 5b, right panel). In summary, these results demonstrated that impairment of HP0860 can cause truncation of the LPS core and thus lead to a significant change of LPS structure in *H. pylori*.

### *HP0860* gene disruption affects protein sorting in OMVs

**Figure 6.**
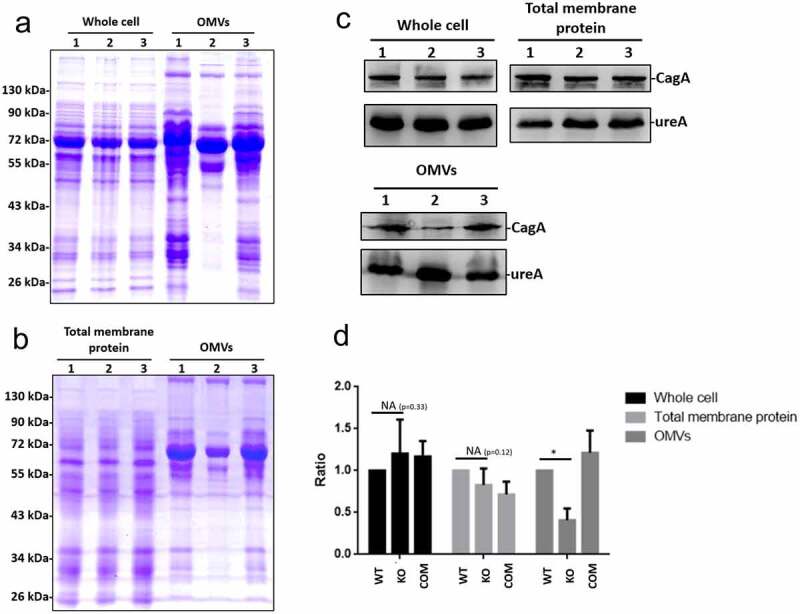
Effects of HP0860 mutations on protein sorting in OMVs. *A*, Protein profile comparison of whole cell lysates and OMVs in different strains of *H. pylori. B*, Protein profile comparison of total membrane proteins and OMVs in different strains of *H. pylori. C*, Comparative Western blots on CagA levels of whole cell lysates, total membrane proteins and OMVs in different *H. pylori* strains. *D*, Statistical analysis of the CagA content in panel *C*. CagA was quantified using ImageJ software and normalized to UreA. (n = 3; *p < 0.05). Proteins from whole cell lysates, total membrane proteins and OMVs were analyzed by 15% SDS-PAGE with Coomassie blue staining, and immune-blotting using anti-CagA and anti-UreA antibodies. M: protein marker; 1, wild type *H. pylori* strain; 2, HP0860 knockout mutant; 3, HP0860 knockout complementary mutant

A recent study targeting at a human oral pathogen *Porphyromonas gingivalis* suggested that LPS structure might direct a protein-sorting mechanism during OMVs formation [48]. Therefore, the protein contents of OMVs collected from different strains of *H. pylori* were subsequently analyzed. Figure 6a and *b* showed that the protein profiles of whole-cell lysates and total membrane proteins were significantly different from those obtained in OMVs, and only a portion of proteins were present or even enriched in OMVs relative to the numerous proteins found in whole-cell lysates and total membrane proteins of the same strains. In addition, the protein profiles of OMVs from HP0860 knockout mutant were also different from those of OMVs collected from wild type *H. pylori* strain. Specifically, the results from immune-blotting analysis revealed that a similar amount of CagA was present in whole cell lysates and total membrane fraction among these strains, and there was no significant difference in CagA levels between wild type *H. pylori* strain and HP0860 knockout mutant (p = 0.33 and 0.12 for whole cell lysates and total membrane proteins, respectively) (Figures 6c and 6d). In contrary, CagA was significantly reduced in OMVs of HP0860 knockout mutant. These results suggested that there is indeed a preference for specific proteins packing into OMVs, which LPS may also play a role in this protein sorting mechanism in *H. pylori*.

### HP0860 knockout mutation has a profound influence on bacterial virulence

**Figure 7.**
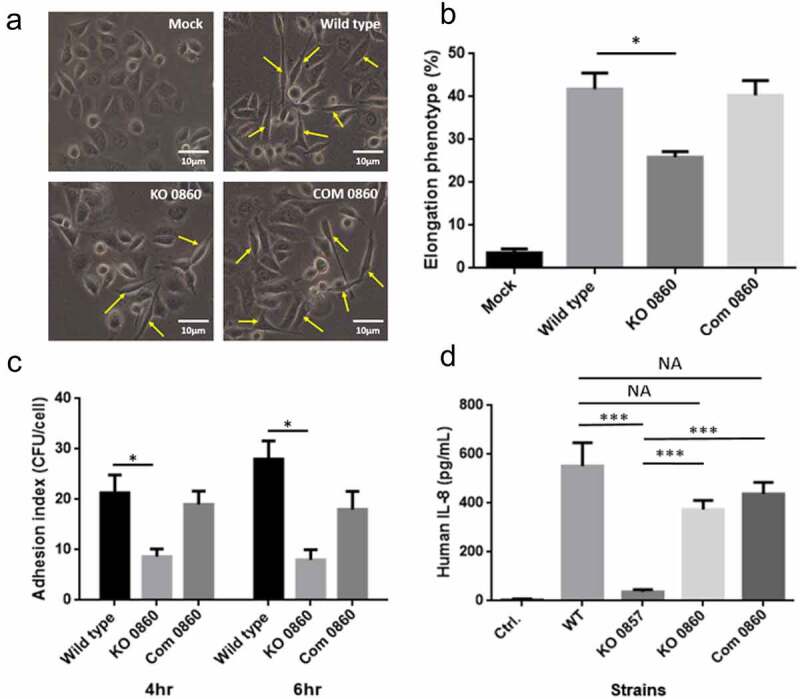
Effects of HP0860 mutations on bacterial virulence. *A*, Comparison of the phenotypic change of AGS cells after *H. pylori* infection. Host cell morphology was documented after *H. pylori* infection by a phase-contrast microscopy with 200 × magnification. AGS cells exhibited an elongated character (hummingbird phenotype) after infection by various *H. pylori* strains were indicated by arrows. *B*, The corresponding percentage of elongated cells was quantified from 10 different 0.25-mm^2^]fields. *C*, Adhesion assay with different strains of *H. pylori* infection. *D*, The result of ELISA assay for detecting IL-8 secretion from infected AGS cells. The IL-8 secretion of AGS cells was quantified after 4 hours of co-incubation with various *H. pylori* strains. Statistical significances were calculated by two-tailed Student’s T test; *p < 0.05, ***p < 0.001. (n = 3). Mock, control group without *H. pylori* infection; WT: wild type *H. pylori* strain; KO 0857: HP0857 knockout mutant; KO 0860: HP0860 knockout mutant; Com 0860: HP0860 knockout complementary mutant

A hallmark of *H. pylori*-infected AGS cells is the development of an elongated and spreading morphological change called the hummingbird phenotype [49]. Thus, to elucidate whether HP0860 knockout mutant would induce AGS cells to express the classical elongation phenotype during infection, AGS cells were infected with various *H. pylori* strains. As shown in Figures 7a and 7b, approximately 42% of AGS cells displayed the typical hummingbird phenotype after 4 hours of co-culture with wild type *H. pylori* strain. Occasionally, elongated AGS cells were also seen in the uninfected cells, but the number was usually less than 5%. In contrast, only 25% AGS cells infected with HP0860 knockout mutant showed the hummingbird phenotype. The proportion (~42%) of hummingbird cells was resorted when AGS cells were infected by HP0860 knockout complementary mutant.

Bacterial adhesion to the surface of host cells is the first step of colonization process and is critical for bacterial virulence. Therefore, the bacterial adhesion assay was applied to explore the adhesion ability of different *H. pylori* strains. As seen in Figure 7c, disruption of HP0860 protein expression caused a significant reduction in the adherence of *H. pylori* to AGS cells compared to that of wild type *H. pylori* strain and HP0860 knockout complementary mutant. These results indicated that the function of HP0860 protein is important for *H. pylori*’s colonization.

Recently, a considerable number of literatures have grown up around the theme of an intermediate product of heptose, HBP, which can be a PAMP and activate NF-κB-related innate immune responses and interleukin 8 (IL-8) secretion in many Gram negative bacteria including *H. pylori*. To test the change of secretion level of IL-8 from AGS cells after *H. pylori* infection, HP0860 knockout mutant and HP0857 knockout mutant (previously constructed in our laboratory) were applied to the cell-based infection study followed by the ELISA assay and the results are shown in Figure 7d. HP0860 knockout mutant, which is expected to still produce HBP in cells, could still induce a comparable level of IL-8 secretion from AGS cells as that caused by infection with wild type *H. pylori* strain, whereas IL-8 secretion was dramatically plunged when HP0857 knockout mutant was applied in the same assay. When tested with HP0860 knockout complementation mutant, it induced a similar level of IL-8 secretion as that by wild type *H. pylori* strain and HP0860 knockout mutant.

## Discussion

In the present study, the essential role of HP0860 protein involved in the synthesis of ADP-L-D-heptose for the assembly of *H. pylori* LPS core was demonstrated. *H. pylori* 26,695 genome contains a gene cluster (*HP0857-HP0860*) encoding proteins involved in ADP-L-D-heptose biosynthesis [50]. Our group has recently reported that the encoded products of *HP0857* and *HP0859* genes, homologues of GmhA and HldD in *E. coli*, respectively, are putative enzymes for ADP-L-D-heptose biosynthesis in *H. pylori*. Both HP0857 and HP0859 knockout mutants of *H. pylori* produced truncated LPS, and exhibited a decreased resistance to hydrophobic antibiotics and a reduced adhesive capacity to AGS cells [29,30]. Thus, to further understand other proteins involved in ADP-L-D-heptose biosynthetic pathway, we selected *HP0860* as our target. *HP0860* gene from wild type *H. pylori* 26,695 strain was cloned and the basic properties of HP0860 protein, and its physiological functions in *H. pylori*, were characterized.

Inspection of the alignment result of HP0860 protein sequence with those of other Gram negative bacteria revealed that HP0860 displays a high degree of similarity with GmhB. HP0860 protein, like its homologues, has three conserved sequences motifs: DXDX(T/V), S/T and (G/S)DXX(N/T)D which compose a core domain of the enzyme’s catalytic active site, suggesting that the function and catalytic mechanism of HP0860 protein may be quite similar to that of GmhB [46]. The molecular weight of a single subunit for the purified recombinant HP0860 protein is about 22.1 kDa, and the native state of HP0860 protein existing in solution appears to be a monomer, which both agree with the biochemical data from the reports of *E. coli* and *M. tuberculosis* GmhB [31,36].

*Glycero-manno*-heptose is not only present in the core of LPS of most Gram negative bacteria, but also found in capsules, O-antigens and S-layer glycoproteins. The biosynthesis of this unique glycan and its derivatives requires a nucleotide activation process, and can be divided into two groups: 1) GDP-D-*glycero*-α-*manno*-heptose biosynthesis pathway, which is represented by the Gram positive bacterium *Aneurinibacillus thermoaerophilus* for glycosylation of S-layer proteins [51], and 2) ADP-L-*glycero*-D-*manno*-heptose biosynthesis pathway in *E. coli* for the assembly of LPS inner core oligosaccharide [34]. It was originally suggested the biosynthesis of D-α-D-heptose can be distinguished from that of L-β-D-heptose by the anomeric specificity of the kinase and the nucleotidyltransferase used. In contrast, the isomerase and phosphatase were considered to function in both pathways without the anomeric preference of the sugar phosphate substrates [28]. However, the results of latest studies indicated that the catalytic activity of GmhB indeed has an anomeric preference for the sugar phosphate substrate, and this preference is dependent on the target molecule in which the final product of the *glycero-manno*-heptose biosynthesis will be incorporated [32]. In *H. pylori*, our data indicated that HP0860 displays a preference for the β-anomer over the α-anomer of D-*glycero*-D-*manno*-heptose-1,7-bisphosphate substrate, which agrees very well with the anomeric specificity of GmhB enzymes shown in *E. coli, Bordetella bronchiseptica* and *Mesorhizobium loti*, which are all involved in the assembly of LPS inner core oligosaccharide [32]. As for GmhB in *Bacteroides thetaiotaomicron*, this enzyme has significantly higher activity with the α-anomer versus the β-anomer, and is associated with the generation of extracellular capsular polysaccharides [32]. Overall, our HP0860 data provide clear evidence which echoes the existence of an anomeric preference of GmhB toward its physiological substrate.

Based on the biochemical data obtained from the *in vitro* enzymatic activity analysis, HP0860 protein was essential for the catalytic reaction in ADP-L-D-heptose biosynthesis. It was reasonable to predict that HP0860 knockout mutations in *H. pylori* might cause LPS truncation. Thus, to more closely simulate the *in vivo* situation, HP0860 knockout mutant was generated to evaluate the effects of *HP0860* mutations on LPS phenotypic changes in *H. pylori*. We found HP0860 knockout mutant indeed displayed a severe loss of LPS core in log phase of bacterial growth, but only showed a partial defect in the synthesis of LPS core in stationary phase. These findings suggest the disruption of *HP0860* gene expression causes the formation of both heptoseless and heptose-rich forms of LPS core in *H. pylori* 26,695 strain. However, this mixed phenotype is restored back to a wild type like heptose-rich LPS structure in HP0860 knockout complementary mutant. A similar phenomenon was also observed in a study that the deletion of *gmhB* in *E. coli* did not lead to a complete heptoseless LPS structure but only caused a partial defect in the synthesis of LPS core. The authors in that study originally proposed the histidinol-phosphate phosphatase activity of a bifunctional protein HisB, which is a homologue of GmhB and is involved in *L*-histidine biosynthesis, might be the candidate activity to at least partially compensate for the function of GmhB for the synthesis of heptose-rich LPS core [34]. Interestingly, the result of an evolutionary study on *hisB* gene has suggested that the ancestor gene encoded a DDDD-phosphatase with a broad substrate range might go through gene duplication event and then finally diverge into two copies of genes: one encodes a histidinol-phosphate phosphatase in the histidine biosynthesis (HisB), and the other encodes a GmhB in the LPS biosynthesis, and both of them have a narrow specificity than that of their ancestor enzyme [52,53]. However, when *gmhB* gene was disrupted in an *E. coli* strain carrying a deletion in the *his* operon, a lack of a complete heptoseless phenotype was still observed [34]. We have tried to use the BLAST program to search for HisB homologous sequences or other possible candidates in *H. pylori* protein database but failed to discover any meaningful protein matched to HP0860, which suggests that the compensating effect of HP0860 knockout mutation found in our current study could not be inferred solely by sequence homology. However, the possibility of the existence of a compensating effect in HP0860 knockout mutant with an additional phosphatase activity cannot be excluded.

Moreover, we also evaluated the effects of HP0860 knockout mutation on other traits. HP0860 knockout mutant had an extended lag phase and a shorter log phase, and became more vulnerable to antibiotic novobiocin. Similarly, Stein et al. also found that the corresponding HP0860 knockout mutant had an attenuated growth compared to its parental N6 strain which is in line with our bacterial growth result despite of the use of different bacterial strains [27]. In addition, HP0860 knockout mutant also showed a dramatic reduction in the adherence ability to AGS cells, and AGS cells infected by this mutant appeared less classical hummingbird phenotype compared to that of wild type *H. pylori* strain. These findings implicate that, in spite of the presence of a compensating effect, a *H. pylori* 26,695 mutant lacking HP0860 seems to be less virulent just like the corresponding mutants in *E. coli* and *S. typhimurium* [28].

To further investigate the role of HP0860 playing in *H. pylori* physiology, small vesicle structures called OMVs which are frequently found on the surface of wild type *H. pylori* were also applied for exploration in this study. The shedding of OMVs by Gram negative bacteria has been observed for a long time; however, it is more recently that their prospective contribution to bacterial virulence has become more widely studied [47,48]. OMVs are usually composed of periplasmic and outer membrane constituents such as LPS, but the presence or absence of inner membrane or cytoplasmic components in OMVs is currently under debate [54]. In the current study, we reported that, in spite of having a slightly different LPS profile, HP0860 knockout mutant contained both mature and immature forms of LPS molecules on the outer membrane surface of bacteria. However, LPS molecules carrying completed polysaccharide chains were less found in OMVs from HP0860 knockout mutant, revealing the existence of a sorting process on the outer membrane of *H. pylori* according to LPS composition or length for packing bacterial components into OMVs. One of the proposed functions for OMVs formation is to promote bacterial survival [55]. It has been suggested that release of OMVs is a novel envelope stress response which allows the bacterium to preferentially pack and eliminate unwanted or mis-structured materials. From this point of view, HP0860 knockout mutant generated OMVs which carried LPS molecules with shorter polysaccharide chains would be advantageous to combat the envelope stress by removing the immature forms of LPS. On the other hand, one study reported that the protein contents in *P. gingivalis* OMVs were affected by modification of LPS structure [56]. Here we also observed that *H. pylori* OMVs were constituted from a limited subset of proteins relative to the whole cell lysates or total membrane proteins of the same strain. The near absence of a virulence factor CagA protein in the OMVs of HP0860 knockout mutant was found in our present study. This phenomenon suggested that the integrity of LPS is required for the selective sorting of some cargo proteins into OMVs, which is correlated with the suggestion provided for *P. gingivalis* that LPS might be involved in an unidentified mechanism which selectively packs certain proteins into OMVs.

HBP in several Gram negative bacteria was found to trigger NF-κB-related innate immune responses after infecting mammalian cells. However, literatures have recently emerged that offer contradictory findings about the genuine PAMP to induce NF-κB activation during host cell infection. Ping et al. recently showed that ADP-heptose but not HBP can be recognized by alpha-kinase 1 (ALPK1) as an effective immunomodulator to elicit an innate immune response in mice [24]. HBP can be transformed to ADP-heptose-7-P by adenylyltransferase in host cells and thus activate ALPK1 to a less degree comparing to ADP-heptose [57]. The most recent work by Pfannkuch et al. also identified that rather than HBP, ADP-heptose is the predominant PAMP in *H. pylori* to activate NF-κB-related responses in infected AGS cells [57]. In the present study, the finding that IL-8 secretion of AGS cells cannot be stimulated by the infection with HP0857 knockout mutant is in line with a previous report [27]. HP0857 protein, GmhA, is the first enzyme in heptose biosynthesis pathway which converts D-sedoheptulose-7-phosphate into D-*glycero*-β-D-*manno*-heptose-7-P. The disruption of HP0857 expression will terminate the final production of ADP-heptose; therefore, HP0857 knockout mutant strain fails to induce IL-8 secretion in infected AGS cells. Interestingly, although the blockage of HP0860 encoding enzyme, GmhB is expected to also affect the final production of ADP-heptose, the infection with HP0860 knockout mutant can still induce significant IL-8 secretion in AGS cells in our current study (Figure 7d). We propose, in accordance with our above results of LPS expression, the loss of function of HP0860 protein due to *HP0860* gene disruption might be partially compensated by other phosphatase activities in *H. pylori* so that the ADP-heptose can still be synthesized in a comparable amount which was also suggested by Pfannkuch et al. [57]

Combining all the data collected, this work has shown that HP0860 is indeed involved in LPS core biosynthesis in wild type *H. pylori* 26,695 strain, and HP0860 knockout mutant with truncated LPS appears to be less virulent. The knowledge acquired provides a new insight to our current understanding of *H. pylori* pathogenesis and the findings also add a new prospect for future drug development against *H. pylori* infection.

## Conclusion

In summary, we reported that HP0860 protein was a phosphatase capable of converting D-*glycero*-D-*manno*-heptose-1,7-bisphosphate into D-*glycero*-D-*manno*-heptose-1-phosphate. The HP0860 knockout mutation in wild type *H. pylori* 26,695 strain caused LPS truncation and influenced the sorting of proteins and LPS to OMVs. HP0860 knockout mutant also showed a flawed growth and was much more sensitive to novobiocin treatment. In addition, less presence of the classic hummingbird phenotype and a lower adherence of bacteria were observed, while AGS cells were infected by HP0860 knockout mutant. Altogether, *H. pylori* lacking HP0860 appeared to be less virulent. Our above findings make GmhB protein a promising target for the development of antimicrobial agents against *H. pylori* infection.

## Supplementary Material

Supplemental Material

## Data Availability

Data sharing is not applicable to this article as no new data were created or analyzed in this study.
