# Supplementary material for: Helicobacter pylori GmhB enzyme involved in ADP-heptose biosynthesis pathway is essential for lipopolysaccharide biosynthesis and bacterial virulence
Source: Virulence. 2021 Jun 14;12(1):1610–28. doi: 10.1080/21505594.2021.1938449 (PMC8204981; doi:10.1080/21505594.2021.1938449)
Supplement: Supplemental Material [file KVIR_A_1938449_SM5557.zip › suppl/supplementary.pdf]

HP0860 knockout mutant; Com 0860: HP0860 knockout complementary mutant.

Supplementary materials

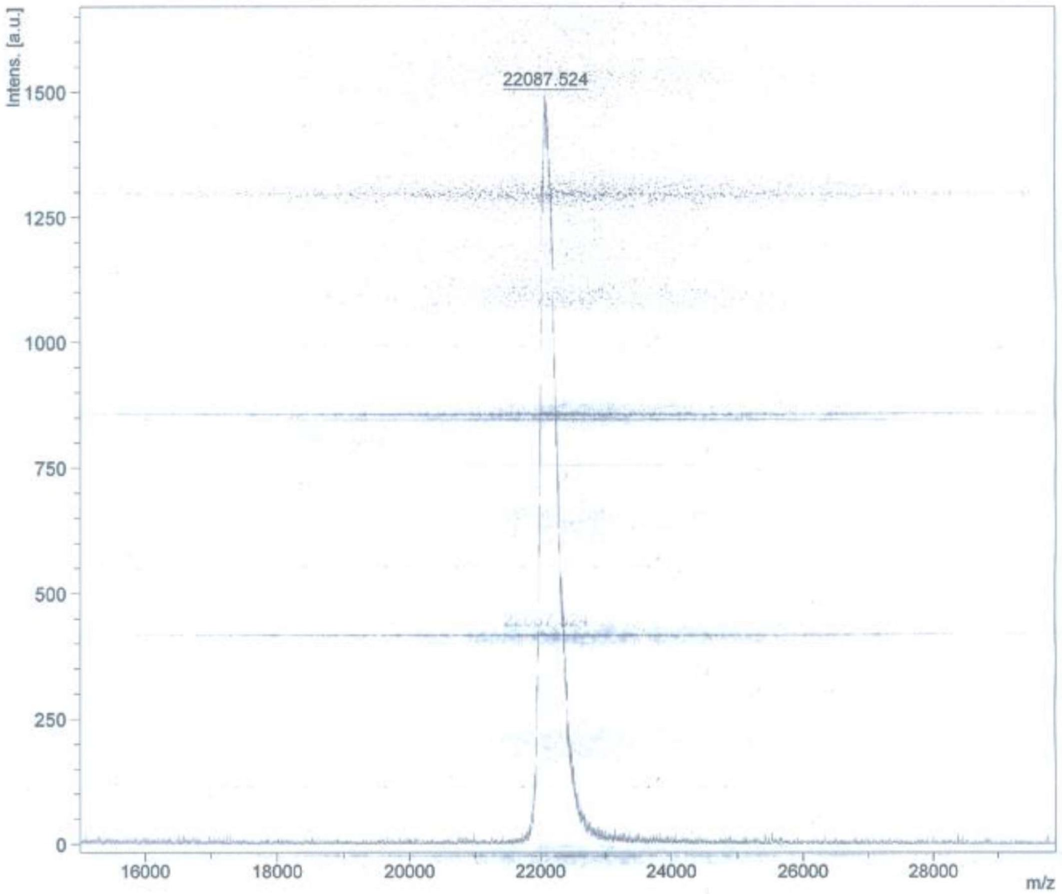

Supplementary Fig. 1 The single subunit molecular weight of recombinant HP0860 determined by MALDI-TOF MS was 22.09 kDa.

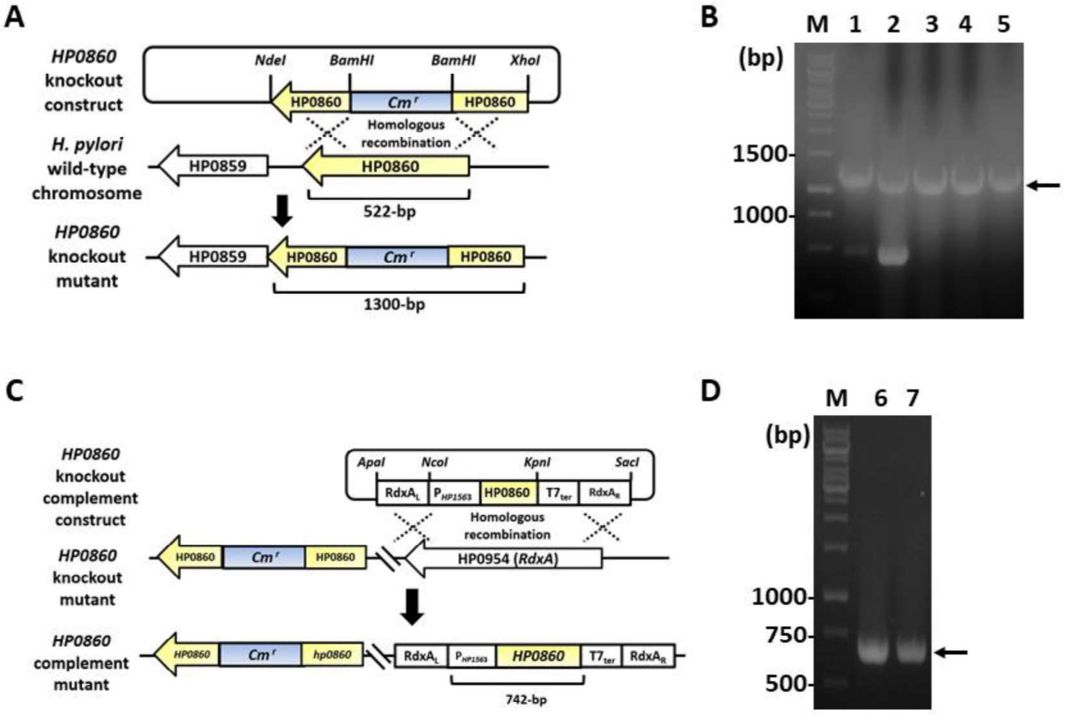

Supplementary Fig. 3 *A,* Restriction map of the *HP0860* genomic region with the insertion of chloramphenicol resistance cassette (Cm^r) into the *BamHI* site to disrupt *HP0860* gene. *B*, PCR amplification from the genomic DNA isolated from HP0860 knockout mutants gave rise to a 1300 bp DNA fragment, confirming the correct construction of HP0860 knockout mutant. *C*, Restriction map of the HP0860 knockout complementary construct showing the *HP0954* (*RdxA*) gene with a *HP0860* insertion. *D*, PCR amplification from the genomic DNA isolated from HP0860 knockout complementary mutants gave rise to a 742 bp PCR product, confirming the correct generation process. M: DNA ladder; 1-5: selected colonies of HP0860 knockout mutant; 6-7: selected colonies of HP0860 knockout complementary mutant.
